# Working Towards Culturally Safe and Sensitive Hospital Social Work Practice in Northern Adelaide, South Australia

**DOI:** 10.1007/s40615-025-02543-1

**Published:** 2025-07-22

**Authors:** Michelle Jones, Luke Cantley, Donna Quinn, Jasmine Bishop, Stacey George, Toni Shearing, Marion Champion

**Affiliations:** 1 University of South Australia, Kaurna Yerta, SA Australia; 2 Flinders University, Kaurna Yerta, SA Australia; 3 Northern Adelaide Local Health Network, Kaurna Yerta, SA Australia

**Keywords:** Aboriginal health, Social work, Hospital, Culturally sensitive and safe practice, Good health and wellbeing, Reduced inequalities, Gender equality, Racism, Social justice

## Abstract

Aboriginal health consumer voices are rarely heard within Australian health systems. Racism is a barrier to accessing health services and improving health outcomes for Aboriginal health consumers. To improve health services and outcomes, social workers from NALHN sought feedback from Aboriginal health consumers on how to improve their practice within the confines of the hospital. Decolonising interpretive phenomenological sequential qualitative research methods were adopted. Non-probability purposeful and convenience sampling was used which was not intended to be representative. Focus group interviews (*n* = 2) and yarning circles (*n* = 4) were held with hospital social work practitioners (*n* = 17) and Aboriginal health consumers (*n* = 5), respectively. Thematic analysis was undertaken by Aboriginal and non-Aboriginal researchers and initial themes brought together. Aboriginal health consumers raised concerns about the stereotypes associated with social work, the importance of establishing a connection through finding out about Nation/Groups and family networks. Aboriginal health consumers requested engagement with family and a recognition of current personal circumstances, not past. Social workers were found to be aware of their privilege and the impacts of colonisation on their practice yet were unsure ‘how’ to put the knowledge they have into practice in the hospital setting. Constraints from the hospital environment were found to limit social workers ability to offer brief, timely, relational, culturally safe, trauma informed social work practice to Aboriginal health consumers. There is a need for the creation of a ‘third space’ within the confines of the hospital. A space where Aboriginal health consumers and social workers can come together assumptions can be challenged, providing opportunity for the development of relational, culturally safe, trauma-informed social work practice.

Concerns have been raised about the cultural safety of Australian health services and their ability to provide accessible and equitable services for all [1, 2]. Institutional racism has been identified as a significant barrier in the delivery of health services to Aboriginal and Torres Strait Islander peoples^1^ [3]. The National Safety and Quality Health Standards (NSQHS) have called for health services and health professionals to partner with Aboriginal and Torres Strait Islander health consumers and improve their cultural competency [4].

Australia’s colonial history infiltrates policy structures and service systems [5]. It is no surprise then that Aboriginal peoples are disproportionately represented in our health systems as health consumers [6]. Aboriginal people’s health is characterised by unacceptable levels of morbidity and mortality. The survival rate of Aboriginal people is lower across all cancer types, 64% of burden of disease among Aboriginal people is due to chronic disease, and one in three Aboriginal youth experienced higher levels of psychological distress compared with one to eight non-Aboriginal youth [7]. Yet despite such health disparities, the Aboriginal workforce within South Australian (SA) Government Health Services remains well below the target of 2% [8].

To rectify this, SA Aboriginal leaders have called for an ‘integrated, cross-discipline, cross-portfolio and Aboriginal-led approach to reforming health programs and practices that are based on what Aboriginal people say’ [7]. Furthermore, leaders continually remind governments that Aboriginal health consumer voices are critical to understanding the impacts of practice on their wellbeing (see National Safety and Quality Health Service Standards [9]).

The hospital social worker explores with the client their social supports, housing, finances, work and spirituality, recognising that these areas also impact on the ability of a patient to recover from their physical ailment and be discharged safely from hospital [10, 11]. Social workers have a history of contributing to colonial practices in Australia through the implementation of welfare policies. Examples include the Stolen Generation and more recently the Northern Territory intervention [12]. Such policies and practices included the forced removal of Aboriginal children and infants which have been responsible for inflicting trauma and loss amongst Aboriginal families and communities [13], resulting in mis-trust toward the profession. Disparities in rates of reports and removals remain. For example, in South Australia, 1 in every 3 Aboriginal children is subject to an unborn child protection notification, compared to 1 in 33 non-Aboriginal children [14]. In addition, most social workers are white Euro-Australian [15], where their power and privilege has been unquestioned and their race invisible. Only recently have social work schools begun to address the invisibility of Indigenous Australians in social work curricula [13] and need for decolonial approaches in social work field education [16]. This study complements and extends previous research focused on social work practice at the intersection of Aboriginal Hospital Liaison Officers and social workers [17–19]. It further explores hospital social work practice as experienced by both the Aboriginal health consumer and hospital social workers in a metropolitan hospital.

This research project is taking steps to decolonise social work practice within the hospital setting. The social work professional association, the Australian Association of Social Workers (AASW), requires social workers have an ethical commitment to culturally competent, safe and sensitive practice [20] and engage in culturally responsive and inclusive practice with Aboriginal peoples [10]. To better understand the intersection of the Aboriginal health consumer and social worker within the hospital, we use the concept of the third space. Coined by Homi Bhabha, a postcolonial cultural theorist, the third space is a space where different cultures intersect and meet, [21] an in-between space. While often a site of confrontation and contestation, it can be an opportunity for differing worldviews to be presented and challenged, moving understandings forward into a space of transformation, identifying the difference but also the common ground [22]. Often used within education settings [23], Homi Bhabha’s concept of hybridity or the third space has been applied by medical anthropologists to the perception of illness, self-diagnosis and power in healing relationships demonstrating applicability to healthcare [24] and in Aboriginal healthcare [25]. Hybridity or the third space centres culture, but no one culture; it traverses the boundaries and interaction between cultures, challenging cultural dominance. The third space has also been proposed to enhance healing and promote understanding about the ongoing impacts of colonialism between Aboriginal and non-Aboriginal social workers and supervisors [26]. Led by a Kamilaroi woman, Sorby et al., the act of social work cultural-clinical supervision in the third space is one that allows for cultural infusion, relationality, deep listening, [26] and learning through ‘hybrid yarning’ [27]. Here, we propose the use of the third space to enhance practice within the hospital setting between social workers and Aboriginal health consumers.

The aim of this study was to develop culturally responsive and inclusive hospital social work practice informed by the voices of Aboriginal health consumers. This paper seeks to answer the following research question: What do Aboriginal health consumers suggest needs to change to support the development of more culturally respectful and safe social work practice in the hospital?

## Methodology and Methods

Our research was informed by both Indigenous knowledge systems and non-Indigenous knowledge systems. The methodologies used were interpretive phenomenology and postcolonial Indigenous methodologies. These methodologies allowed for the exploration of peoples’ subjective experiences of the world, recognising our experiences are socially constructed, interpreted [28] and that we each attribute meaning to our experience in the world [29]. The use of postcolonial Indigenous methodologies through the use of yarning circles allowed for challenging colonial practices and deficit thinking about First Nations peoples, seeking transformation and social change [30]. Qualitative research methods including focus group interviews with social workers and yarning circles with Aboriginal health consumers were undertaken. Focus groups are a form of in-depth interviewing and were selected as the social workers worked together and shared a common relationship to the topic [31]. The group format allowed the research to capitalise on the synergy gained from sharing ideas [32]. Focus groups were facilitated by an Aboriginal social work student researcher and a white Anglo-Celtic fifth generation colonial settler social work researcher. Yarning is an Indigenous conversational process that involves the telling of stories and sharing of information; it is culturally ascribed and cooperative [33]. Yarning was used due to its relaxed approach and ability to create a culturally safe environment [34, 35]. To respect cultural protocols, both a male and a female Aboriginal researcher undertook yarning circles to identify the areas for improvement to hospital social work services.

### Sampling, Participants and Recruitment

Non-probability sampling was adopted including a combination of purposive and convenience sampling. Participants were purposefully selected based on their experiences, expertise and cultural backgrounds [36, 37]. This study was not intended to be representative, and there were no fixed recruitment ratios or target number of participants. Convenience sampling [37] was used for the yarning circles as Aboriginal health consumers were recruited whilst attending the Aboriginal health services for an appointment. All Aboriginal health consumers attending the health service on the day of our visits were invited to be involved in the study. Participant numbers reflect those whom consented to be involved in the study. This may have limited our Aboriginal participant numbers as our response rate depended upon the number of appointments booked on the days we attended the Aboriginal Health Service and the availability of Aboriginal health consumers before or after their health appointment. As the research was qualitative, we sampled until reaching saturation point, where no new meanings are revealed [37]. This can be reached in quite small samples with homogenous sample populations and narrow research questions [38]. Saturation point was used to guide sample size as not to contribute to the ‘research burden’ placed on Aboriginal and Torres Strait Islander peoples [39] demonstrating respect. In addition, the research was conducted during and just following COVID where Aboriginal and/or Torres Strait Islander communities were closed to health research [40].

There were two participant groups: Aboriginal health consumers and social workers. The Aboriginal health consumers were of Aboriginal and/or Torres Strait Islander background, spoke English and have either needed or received a service from social work in relevant hospitals. The health consumers were not required to have had attended a social work appointment; they may have been referred but did not attend or it may have been a family member who received social work services. Aboriginal members of the local Aboriginal Health Consumer Reference Group were invited to attend, and posters with information about the aims of the research were placed in the waiting areas of the local Aboriginal health services. Information sheets and consent forms were available in the waiting area and provided before attending the yarning circles and/or provided on the day. Administrative and health practitioners at the Aboriginal Health Service were advised of the research visits and invited patients to consider participation in the study. Lunch from an Aboriginal-owned and operated catering business was supplied to participants as was the provision of a supermarket gift voucher in recognition of the participant’s time. Five yarning circles were scheduled across two Aboriginal Health Services in metropolitan north-west Adelaide; one was cancelled due to flooding. Four yarning circles were held, and in total, there were five Aboriginal health consumers in attendance: three females, two males and no non-binary participants. Sample demographics are summarised in Table 1.

**Table 1:** Sample demographics

Population/Participants	Demographics			*n*
Yarning Circles	1		0	
	2—cancelled due to flooding		-	
	3		2	
	4		1	
	5		2	
				5
Focus Groups	1 at Lyell McEwin Hospital		10	
	2 at Lyell McEwin Hospital		7	
				17
Total				22
Health Consumers				5
	Gender	Female	3	
		Male	2	
		Non-binary	0	
	Cultural background	Aboriginal and/or Torres Strait Islander	5	
Social workers				17
	Gender	Female	15	
		Male	2	
		Non-binary	0	
	Experience	Qualified SW	17	
		Student SW	0	
	Cultural background	Aboriginal and/or Torres Strait Islander	0	
		Australian	7	
		Western (European)	4	
		Eastern (Asia, Middle East)	6	
Total				22

The social work teams were across two metropolitan hospitals in one local health region, and their social work students were invited to be involved. The senior social worker distributed an email from the researchers to the hospital SW staff inviting their attendance and including the information sheet and consent form. Focus group interviews were held to accommodate rosters across two hospitals, ensuring services could be maintained. Authors 1 and 2 engaged the hospital social work team in focus group interviews to understand the status of their current engagement with Aboriginal health consumers. For example, asking social workers to reflect upon the knowledge, skills and tools they draw on; their level of comfort; what works well and what leaves them feeling they have not supported the Aboriginal health consumer. Two focus groups were held with a total of 17 participants, and demographics of the sample are provided in Table 1. Most social work participants identified as female (*n* = 15) and Australian (*n* = 7). While blunt, to reduce the identifiability of participants, we have used the broad categories of Western and Eastern to report the cultural backgrounds of the social workers.

### Data Collection and Analysis

A semi-structured interview schedule (Table 2) guided the conduct of the yarning circles and focus groups, and demographic data was limited to Aboriginality (not language group) and gender. Focus group interviews (conducted by authors 1 and 2) and yarning circles (conducted by authors 2 and 3) were voice recorded and transcribed. Thematic analysis [41] of focus group interviews and yarning circles was undertaken by authors 1, 2 and 4 using NVivo 12 qualitative data analysis software. Analysis was undertaken independently, and then the three researchers brought their themes together and consensus was reached. Adopting interpretive phenomenology and postcolonial Indigenous methodologies meant that ‘a belief or claim from a culture one does not understand is consistent and correct’ p.39 [30]. In practice, this was actioned by privileging (or placing importance) on the Aboriginal researchers’ interpretations during analysis and reporting of the yarning circles.

**Table 2:** Semi-structured interview schedules

Focus group	
1	What is the nature of and hours of the services that social work offers? Do you get to see all the people who are referred to you?
2	How do you get referrals or how do you get your clients? Are your referral processes for Aboriginal clients any different to non-Aboriginal health consumers?
3	We understand that you have undertaken a cultural reflection process, using an audit tool, what surprised you about this process and the outcomes?
4	What knowledge and tools do you think could best inform your social practice when engaging Aboriginal health consumers?
5	How do you build a connection with your client? How do you know if your client is Aboriginal and/or Torres Strait Islander?
6	Reflecting on your social work practice, what do you believe works well to engage NALHN Aboriginal Health consumers?
7	In what specific clinical assessment situations does your social work practice with NALHN Aboriginal health consumers leave you feeling like the consumer engagement was not effective or provided little or no support to the client?
8	When do you feel comfortable in your engagement with Aboriginal health consumers and when do you feel uncomfortable in your engagement with Aboriginal health consumers?
9	What are you worried about in your engagement with Aboriginal health consumers?
10	What knowledge or skills do you think you need to develop to improve engagement with Aboriginal health consumers?
11	How do you want Aboriginal health consumers to experience the social work services at NALHN? What do you think an ‘ideal scenario look like’? How do you know? How will you know if your engagement is on track?
12	Aboriginal Researcher asked: As an Aboriginal man self-determination is critical to the provision of culturally safe services for Aboriginal people. How do you feel that you respect the self-determination of Aboriginal health consumers within your social work practice?
Yarning circle	
1	What do you know about the hospital social workers and what they do? Have you ever been to see a social worker in hospital [which service]? Have you been told to see one but did not go? What stopped you from going?
2	What are your opinions about hospital social workers?
3	If you have seen one what are your experiences of seeing the hospital social worker or what stories have you heard from others about visiting a hospital social worker? Do you feel culturally safe when you visit a hospital social worker?
4	What are the hospital social workers doing well? What makes it ok when you visit a hospital social worker?
5	What do you think needs to change so that you would feel culturally safe to use the hospital social work services?
6	What could be done better to improve your experience when seeing a hospital social worker? Do they need to change the environment? What they say to you? How they communicate with you? Do they need more cultural understanding?
7	How do you know when you get a good service from the hospital social workers?

### Research Team

The cultural backgrounds of the research team (and authors) included four Aboriginal researchers (authors 2, 3, 4 and 6) and three Anglo-Celtic colonial settler researchers (authors 1, 5 and 7).

### Ethics

The metropolitan hospitals Aboriginal Health Consumer Reference Group were involved in the design of the study. This study was approved by institutional (14,383), Aboriginal Health (04–20-907) and university (4398) National Health and Medical Research Council sanctioned human research ethics committees. All participants consented to be involved.

## Findings

**Fig. 1:**
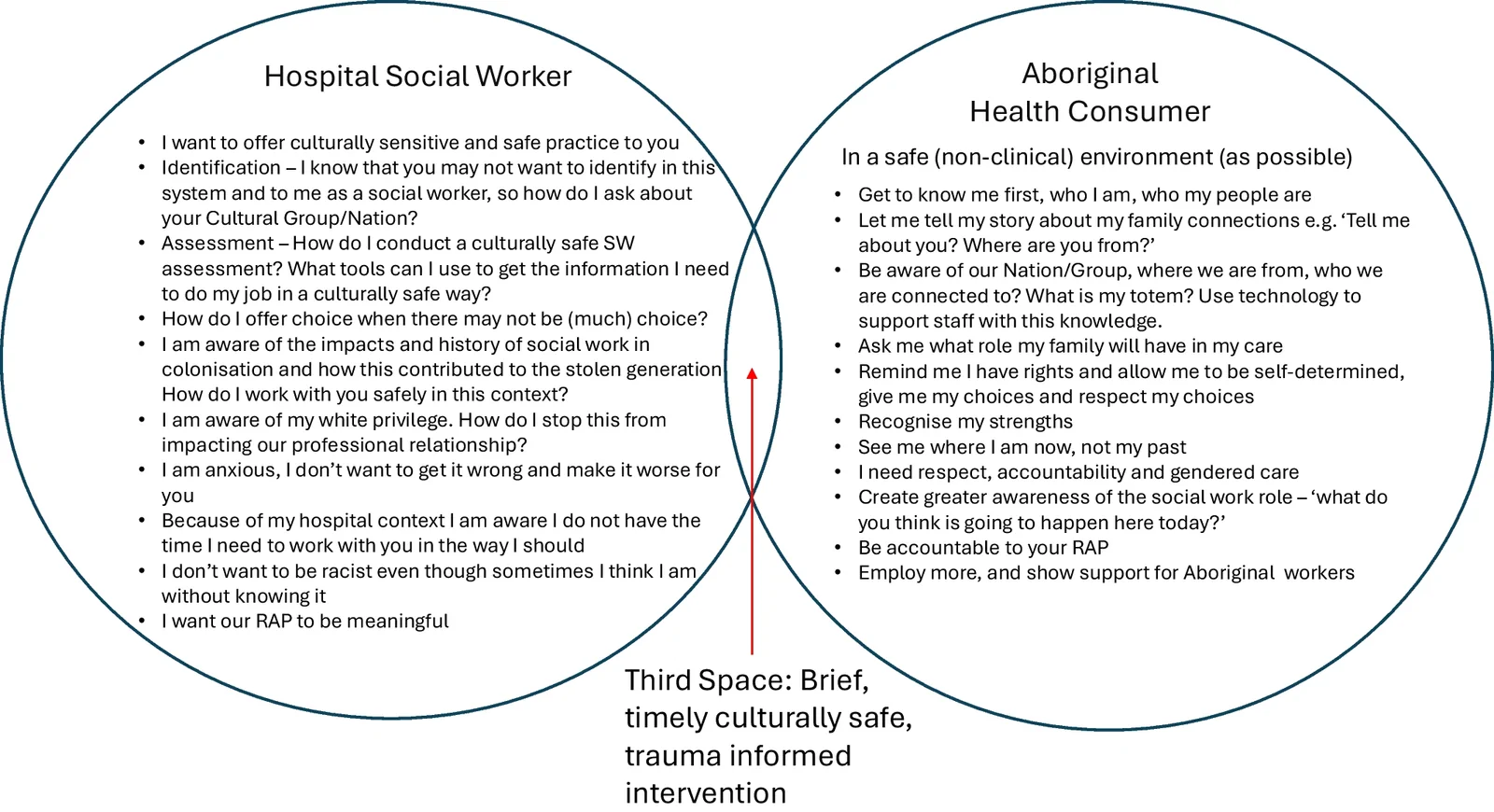
The third space

While not conducted first, the findings from the yarning circles will be presented first as a means of privileging the voice of the Aboriginal health consumers. Due to the small numbers of participants and to protect privacy, the gender of the participants and location of the yarning circles are not reported with the quotes. The themes arising are pictorially represented in Fig. 1.

### Yarning Circles with Aboriginal Health Consumers

The following themes were identified, and quotes have been used to illustrate the themes.

#### Create Greater Awareness of the Social Work Role

All Aboriginal health consumers involved in the yarning circles reflected on the negative stereotypes of social workers, whether it be as a profession in relation to the historical (and contemporary) role within the Stolen Generation or infant removals, or contemporary stories, and experiences of racism within services provided to themselves or others. An Aboriginal health consumer stated:

there’s such a stigma around social workers, so people think ‘Social workers, I’m in trouble. Social workers, there’s trouble.’ Instead of creating knowledge of what … social workers are there to do.

This quote was interpreted as recognising the strong links between the history and stigma of a social worker’s role in Aboriginal health consumers’ lives, and the ever presence of colonial history and policies. Health consumers expanded on their frustrations with social workers: ‘they [SW] build your anger up, … they make you want to get out of [hospital] bed and flog them’. In addition, another Aboriginal health consumer expressed concerns with the generalisations or stereotypes that social workers make: ‘they [SW] always categorise us, as in we don’t look after ourselves, and we’re either alcoholics or drug addicts’ which may result in not attending the appointment: ‘if someone has an appointment with social work, 90% of the time they won’t show up because they are fearful of what’s going to happen…instead…you [SW] ask them “what do you want to get out of today’s session? What do you think I’m [SW] here to do for you?”’ They suggested that making the SW interview more personal for the Aboriginal health consumer will allow for a conversation about the social work role.

#### Get to Know Me, Establish a Connection With Me

In the yarning circles, Aboriginal health consumers asked that social workers be aware of their Nation or Language Group, where they are from and who they are connected to: ‘Ask perhaps “What my totem is?” and use technology to support this.’ The Aboriginal health consumers suggested social workers get to know who they are first and who their people are. Another Aboriginal health consumer stated:

Yeah, probably just have a conversation with them about them, not necessarily about what the situation is or the potential risk factors of whatever it is they’re reading from somebody’s file. To me, it would be, ‘Have a conversation, get to know who I am before you start digging deep’.

Another Aboriginal health consumer asked to be provided the opportunity to tell their story and family connections so the social workers could open their conversation with questions or statements: ‘Tell me about you. Where are you from?’ An Aboriginal health consumer stated:

The best thing with [AIATSIS] Aboriginal map in your social worker’s office, which is under your RAP [Reconciliation Action Plan]... Just ask, ‘Where do you come from?’ It opens engagement when you start yarning.

The Aboriginal health consumer suggested the social workers use technology to find the knowledge and information about their Nation or Language Group and be self-sufficient, taking professional responsibility for building this knowledge.

#### Remind Me I Have Rights and Allow Me to be Self-determined

Given the stigma and stereotypes about social workers within Aboriginal communities, an Aboriginal health consumer suggested creating a greater awareness about the social work role. So, providing information about:

Ask them: ‘What do you think’s going to happen here today?’ So, then you can [clarify the purpose and reduce their anxiety] straight away. ‘Ohh, that’s not true.’ ‘This is what we’re actually here for.’

Another Aboriginal health consumer asked that Aboriginal health consumers be reminded of their rights, and the social workers be reminded that Aboriginal health consumers should be self-determined within the health system: ‘We are supposed to be living in a time of self-determination, self-management and empowerment.’ Where Aboriginal health consumers are provided with choices and their choices are respected as part of their healthcare within the hospital: ‘they’ve got to treat us with respect. And sometimes a lot of these fellows here [social workers] will think we should be grateful they’re doing us a service.’ Another Aboriginal health consumer stated:

You’ve already got hundreds of things going on. Social worker is the least of the problems. [The patient] don’t wanna have a yarn with you [social worker] right now. So, it’s a matter of [the social worker] probably popping your head in [and stating] ‘I’m going to come back and see you when you’re feeling better’ or, taking the clinical side of it out of it. So, finding a space where you can have a chat that’s not in that same space, they’re obviously either having a traumatic event or there’s already had something else going on.

This quote was interpreted as recognising both the importance of both the timing of the social work engagement as well as the location or space suggesting, if possible, not in the clinical environment.

#### Ask Me What Role My Family Will Have in My Care

Aboriginal health consumers also asked social workers what role they want their family to have in their care, and so looking more broadly at: ‘Who are the people in their family and what role they might have?’ An Aboriginal health consumer stated: ‘you want to cope because I do spend [a lot of] time in hospital, the operations and none of my family could go up and see me. So, I was just talking to the cleaners.’ This Aboriginal health consumer reflected on the importance of family and how lonely they were without the support of their family to visit them during their hospital stay.

#### Recognise My Strengths Where I Am Now, Not My Past

An Aboriginal health consumer reflected on the experience of a family member having their second child at the hospital, when years before their first child had been removed from their care. The Aboriginal health consumer stated:

[my family member] was a little bit confronted because stuff that’s on her file, she hasn’t even spoken about or looked at for 20 years, and this was directly brought up with her when she had her children. … She wouldn’t go back and see the social worker. She wasn’t comfortable with having any other conversations with them.

Aboriginal health consumers asked to be seen where they are now, not looking historically at their file at their past and making generalisations about who they are now. Aboriginal health consumers asked to be seen in the now and for the social worker to recognise the change and improvements they had made in their life over time (e.g. leaving a violent relationship). Social workers were encouraged to pay attention to the present, the now.

#### I Need Respect, Accountability and Gendered Care

Aboriginal health consumers asked to have their cultural needs respected and ensure accountability processes were built in. This included being provided with gendered care; a female Aboriginal health consumer, who had worked as a nurse, stated:

Muslim men come in [to the hospital], we … change the roster for a man to nurse that person. But when an Aboriginal man comes in and I’ve [female Aboriginal woman] got to nurse him and I know I’m not allowed to and I ask, they don’t change me. So that person can … make me sick. They [the hospital] don’t care.

This Aboriginal health consumer reflected on her experience of the double-standards as an Aboriginal female nurse being asked to care for an Aboriginal man, where she recognised the cultural inappropriateness of this. This example highlighted the importance of the recognition of men’s and women’s business within Aboriginal communities and how this must too be respected.

#### Be Accountable to Your Reconciliation Action Plan (RAP)

An Aboriginal Health consumer asked hospital social workers to be accountable to their RAP. They stated: ‘Because if you read your RAP plans…You must have resources, you must have Aboriginal flags on your desk. “Where’s your accountability?” You don’t have them…if you were to have simple things, then things would work better.’ This Aboriginal health consumer reflected on the important role the RAPs can have when they are fully enacted within the organisation to provide a culturally safe environment for Aboriginal peoples.

#### Employ More and Show Support for Aboriginal Workers

Finally, one of the most critical points made by all Aboriginal health consumers was the importance of employing Aboriginal workers/social workers and showing support for Aboriginal colleagues. An Aboriginal health consumer stated:

And see that’s in your mob’s funding. … All hospitals get funded for two ALOs [Aboriginal Liaison Officers], one male and one female…You get the money, but you still don’t employ them. So once again, accountability.

This Aboriginal consumer expresses exacerbation with the hospital and the health system, knowing that funding for two ALOs is available and yet there are often gaps in employment of Aboriginal staff.

### Focus Group Interviews with Hospital Social Workers

Two focus group interviews and one interview were held with social workers and the social work senior manager respectively. These were held separately to support the social workers to speak freely about their experiences. The following themes were identified.

#### I Want to Offer Culturally Sensitive and Safe Practice to You

In a focus group, a social worker stated: ‘Just being a social worker can be a trigger.’ Underpinning the social workers comments is care for providing a culturally safe service and awareness of the power and privilege they have by nature of their position and being non-Aboriginal. In another focus group, a social worker stated: ‘we might have our ALO called and [I’d] say, “look I’ve got this person, this is the issue, is there something we can do to complement what you’re doing?”’ Hospital social workers in this study reported being aware of ALO and the importance of them in providing culturally safe care.

#### Identification—How Do I Respectfully Ask About Your Nation or Group?

Hospital social workers described how on most occasions Aboriginal health consumers have already been identified within their administrative system aligning with best practice guidelines [42]. However, this was not always the case. A social worker recognised some Aboriginal health consumers may not be ready to identify as Aboriginal:

sometimes the [Aboriginal Health Consumer] might identify but may not have explored themselves, and so, the thought of having, an ALO might be a bit confronting for them. So, I think constantly asking those questions and making sure we’re on the same page is really important when we’re working with Aboriginal or Torres Strait Islander patients.

Another hospital social worker commented that sometimes, Aboriginal health consumers prefer not to take-up services from Aboriginal staff to protect their privacy.

#### How Do I Conduct a Culturally Safe Psychosocial Assessment?

Hospital social workers talked generally about the importance of the assessment process to their practice and questioned: ‘How they conduct a culturally safe social work assessment and what tools they have available to them to get the information needed in a culturally safe way.’ Another social worker stated:

if there was some sort of assessment tool that was maybe designed and put together by Indigenous people, if it was just a small one [in addition to] our normal psychosocial assessment tool.

The hospital’s social workers were aware the current assessment tools were inadequate and recognised the importance of having culturally safe psychosocial assessment tools to use as part of their usual practice to support Aboriginal health consumers.

#### How Do I Offer Choice When There May Not Be (Much) Choice?

Hospital social workers recognised the importance of offering choice and talked about how they can offer choice to Aboriginal health consumers when there may not be a lot of choice available. A social worker stated: ‘But unless there was a safety risk or some sort of duty of care that superseded, we might get involved in the background. But otherwise, if they don’t want to be seen by social work, then we generally can’t get involved.’ This social worker reflected, on most occasions, Aboriginal health consumers are provided the option to have a social worker involved.

#### How Do I Work With You Safely in the Context of Colonisation and the Role My Profession Has Played?

Hospital social workers reported their awareness of the impacts and history of colonisation and social work’s contribution to the Stolen Generation:

We do a lot of the learning about the true history and the way Aboriginal people have seen their history and have recorded their history. I’ve never had any training which actually says, ‘okay so this is how you’ll work with Aboriginal people.’ When they come in, you understand all this history now, and why they’ve come to this point; now what do we do?

In this quote, the social worker reflected on how this knowledge was not translated to practice and applied to what this means for how they, as a social worker, work with Aboriginal health consumers. Another social worker stated:

the dilemma and the internal conflict I have around recognising the Stolen Generation and the … institutional racism and then our role as a social worker in comparison with statutory social workers…we’re offering our role as support for families. But in fact, in the background we also have that statutory responsibility [to report suspicion a child or young person may be at risk]. I find that really conflicting and knowing there’s history of trauma that sits there and has for many generations.

Again, the hospital social worker shares their awareness of the ongoing impacts of colonisation, in particular the role of the hospital social worker within this. Recognising while they are not social workers working for Child Protection, they still have a statutory responsibility to report their suspicions of risk of harm to a child.

#### How Do I Stop My White Privilege Impacting Our Professional Relationship?

Hospital social workers were aware of their white history and the resultant privilege afforded to them. They questioned how they work to stop their white privilege from impacting their professional social work relationship: ‘I don’t know if it’s conscious or subconscious fear of the fact, I’m white, I’m privileged. Am I going to re-trigger a trauma, [in what] I am going to do, or say?’ This hospital social worker raised concerns about the impacts for Aboriginal health consumers of who they are as a white social worker and all they represent.

#### I’m Anxious I Will Get It Wrong and Make It Worse For You

Another hospital social worker in the focus group interviews talked about being anxious when needing to implement their statutory powers under Sect. 41 of *The Children and Young People (Safety) Act 2017* (SA). The social worker stated: ‘it is really awkward, and I feel very anxious a lot of the time going in to speak to a family I know may be heading down the road of a likely safety plan or a Sect. 41 removal at birth.’ The social worker wanted to ensure the safety of the infant or child; however, they did not want to get it wrong or make it worse for the Aboriginal health consumer. In the following rather lengthy quote, another social worker, working in the maternity ward, reflected on the trickiness of balancing their mandated role with being able to provide a supportive culturally safe service:

we are mandated [to report suspected child neglect], and sometimes has massive cultural repercussions as well, and it can impact the service, so especially if there’s domestic violence or child abuse, straightaway for most of our patients referred, there are big triggers for our patients. So, I don’t think we’ve, as a team or as society [or profession] been given enough training in terms of how to manage that specifically, the ‘Family Safety Framework’ says, if a patient identifies as Aboriginal or Torres Strait Islander, these are the questions or things to consider. But then it’s ‘how do you incorporate that into your assessment while making sure you’re still doing your DV risk assessment?’, and trying to provide a safe outcome for the patient, knowing they’re also in this cultural setting…

Locating themselves in the health system, this social worker shares the complex interplay when making a professional judgement. The social worker questions their ability to respect Aboriginal cultural practices and at the same time ensure that the ‘family safety framework’ is implemented. In attempting to resolve these ethical conflicts, a social worker reflected on the importance of co-working with their Aboriginal colleagues. They stated:

We had a system in Women’s Health before AMIC [Aboriginal Maternal Infant Care] was defunded where we would kind of co-work a case. The social worker would complete the mandatory notifications required and have those kinds of conversations. Leaving the AMIC practitioner to have more of a supportive [cultural] role. They [AMIC practitioner] didn’t have to cross that boundary and go into that sort of legal kind of mandatory notification.

Co-working was a way the social worker reconciled their institutional role as a social worker with their ethical commitment to providing culturally safe and sensitive practice, by ensuring the cultural safety was provided by another practitioner.

#### I Am Aware I Don’t Have the Time I Need to Work With You in the Way I Should

Hospital social workers were aware of the constraints of the hospital environment on their social work practice, such as time. A hospital social worker stated:

if we were to work with Aboriginal patients and be completely culturally appropriate [we] would probably spend more time talking [about what is] important in their life, but we just don’t have the time and the capacity to do that as thoroughly as we probably could. It’s a tricky one.

The social workers reported not having the time to work with Aboriginal health consumers in a culturally respectful relational way to build trust and strong relationships. Another hospital social worker stated: ‘But that’s something obviously with Aboriginal people…, they’re more storytellers. That can be hard when you’re under time pressure, to give them time and give counselling that fits their needs.’ In this quote it is evident the social worker was aware of the importance of spending time to engage with Aboriginal health consumers to develop trust and relational ways of engaging.

#### I Don’t Want to be Racist, Even Though Sometimes I Think I Am Without Knowing

Hospital social workers wanted to avoid being racist, even though in their social work practice, they think sometimes they are being racist without knowing it. A hospital social worker stated: ‘you don’t want to offend Aboriginal health consumers.’ Then a female social worker commented:

I went through a spate of having a lot of Aboriginal patients and finding sometimes the men won’t even look at me, won’t talk to me, can’t engage, but it’s not every Aboriginal male that comes in either…the shock of how limited their trust is towards me, a white [female social worker] person. So, I don’t think there’s enough training on how to best approach that.

In this quote, a female social worker admits her shock in the recognition of the potential need to consider gendered care for Aboriginal patients and calls for skills in how to respond.

#### Aboriginal Workforce

Social workers talked about working closely with ALOs whenever possible and appropriate. They also noted there were times when it was not possible, and they had to ‘go it alone.’ One hospital social worker stated:

because we don’t have a million ALOs available at our beck and call, maybe it is them educating us and saying, well when we’re not around, when we’re all sick, that’s when you go and see a patient, these are some extra things you can add to your social work psychosocial assessment that could help guide you, because they’re not always going to be available.

This social worker called for the ALOs to provide cultural training to the social workers to upskill them. The hospital social work manager talked about the hospital’s workforce plan and requirements to increase the number of Aboriginal staff across the organisation asking: ‘How can we attract more Aboriginal people to our workforce? How can we keep and sustain Aboriginal people in our workforce?’ The social work manager was aware of the importance of Aboriginal people providing services to Aboriginal health consumers. To this end, they were seeking support to ensure the health service could attract and retain Aboriginal social workers.

#### Confusion about the Social Work Role

The social workers talked about the difficulties of working inter-professionally and how on occasions their colleagues make inappropriate social work referrals: ‘we feel we are getting referrals, sometimes not necessarily because it is a social [problem/issue] it’s because other staff in the hospital don’t know what to do.’ Sometimes the social worker feels ‘set-up’ by their colleagues, for example, a clinician saying to the patient ‘social work will get you a house.’ A social worker stated: ‘medical staff don’t understand … there’s nothing out there. … you are basically giving bad news… I can’t do anything.’ Again, in another focus group, a social worker stated: ‘you’ve just promised this family this; we can’t do that.’ In their engagement with Aboriginal health consumers, the social workers felt they were constantly giving ‘bad news’ as they were unable to provide access to the services other staff had promised on behalf of the social worker. The reduction in community services impacts the range of services social workers can refer Aboriginal health consumers to. Social workers are being squeezed on both sides through staff making promises to Aboriginal health consumers about accessing services, without knowing there are fewer services available for social workers to refer patients to.

#### System and Service Pressures

Social workers reported their frustration with the systems, stating: ‘We don’t get to do what needs to be done just due to the systems and structures. I feel … we do the bare minimum, so ensure they’re safe, assess vulnerability, make sure services and supports are in place, but that’s where our role finishes.’ Another social worker spoke about the demands and health service pressures:

we know our work in the ED has tripled in referrals and we have got the data to prove that. We know it’s much more complex work. We’ve got the data to prove that. We have got more complex demographics and complicated multiple layers of health issues. …NDIS for example.

So, while referrals and cases have increased in complexity, there has not been a corresponding increase in staff to support the demand. In addition, COVID resulted in a loss of experienced hospital social workers, which means it is difficult to match social work experience to the level of case complexity. The social workers speak to the frustrations with systems they feel limit their ability to provide high-quality patient-centred care.

## Discussion

This research shed light on the experiences of both Aboriginal health consumers when seeking hospital social work services and social workers’ experiences in the provision of services to Aboriginal health consumers within the hospital environment. Aboriginal health consumers spoke of the importance of building connection, finding out about their family and group or nation; also, the importance of listening to the person and allowing self-determination such as gendered care. The social workers shared their anxieties about engaging with Aboriginal health consumers. Importantly was the concern, by nature of their power—both white privilege and professional power—that they may contribute to experiences of racism. Most social workers reported they knew the past and ongoing impacts of colonisation but did not believe they had the skills, tools nor the environments to provide culturally safe care to Aboriginal health consumers. A paucity of culturally safe tools remains an issue in social work. In relation to securing Aboriginal foster carers, Bromfield et. al. [43] argue assessment processes are based on Anglo-European, middle-class values and parenting standards, and Vance et. al. [44] examine how culture might be embedded into clinical assessment, formulation and treatment. Cultural safety cannot be achieved through knowledge and passive awareness alone. Personal attributes, knowledge, skills and the approach a non-Aboriginal health worker takes are important including strategies of relinquishing power and control, humility and honesty, reciprocity and co-working [45]. Hospital social workers need to engage in active decolonial action through sustained accountable practice, prioritising Aboriginal ways of knowing, being and doing.

**Fig. 2:**
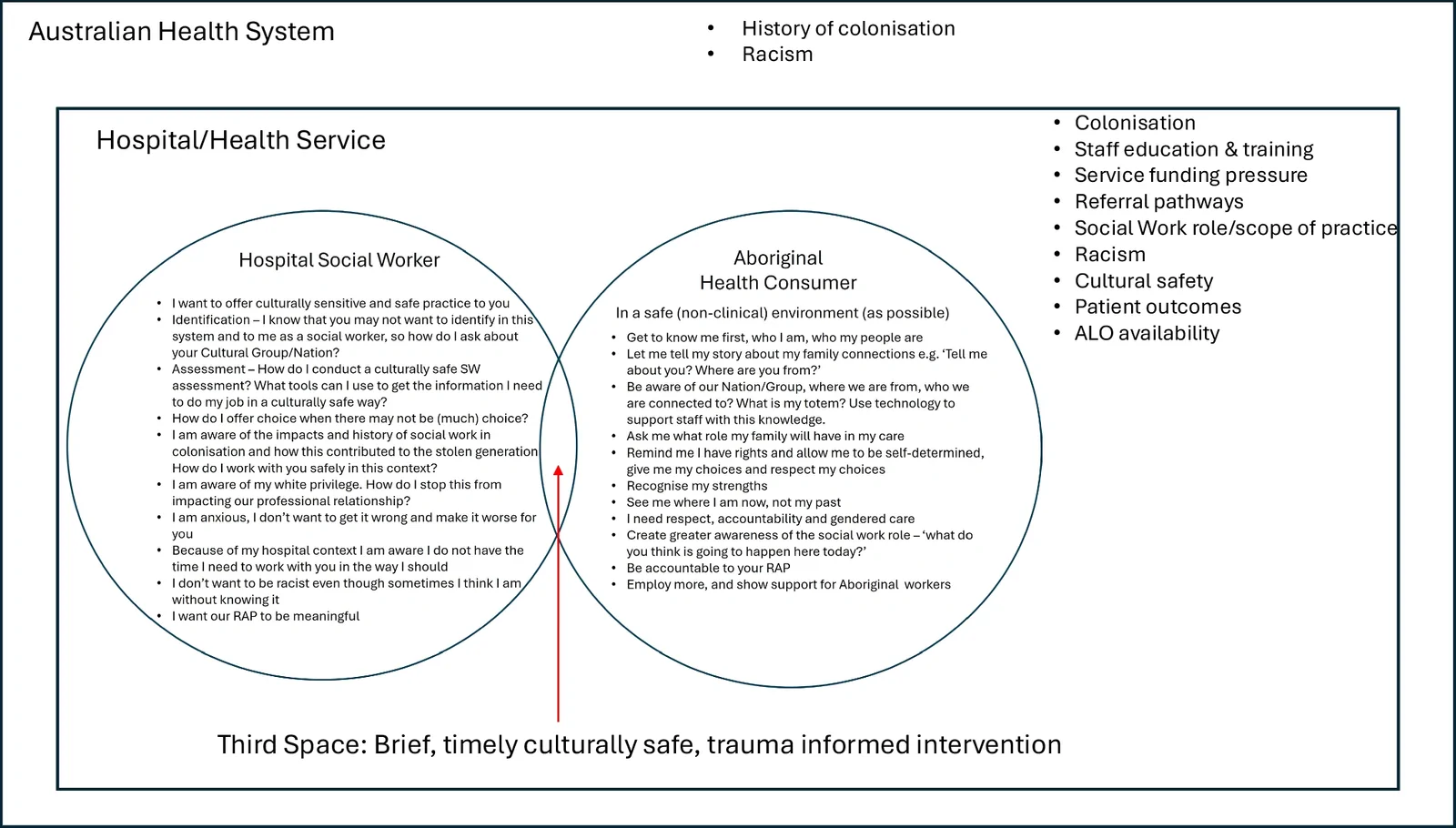
Context for the social work intervention

Studies have found that both individual and organisational contexts impact health professionals’ practice in Aboriginal health [46]. To locate these findings within place (hospital) and space (health system), they have been pictorially represented (Fig. 2). This allowed us to identify and articulate the additional constraints on the interaction resulting from both the hospital and the Australian health system. We found both social workers and the Aboriginal health consumers recognised the constraints of working for a hospital within a health system which both carry their own histories of colonisation and systemic racism [47]. The hospital environment was seen to shape and contribute to the interaction between the SW and the Aboriginal health consumer through service funding pressures, level of staff training, scope of the SW role, availability of ALOs and referral pathways. At the intersection between both the social worker and the Aboriginal health consumer—where they overlap—is what we have identified as the need for a brief, timely, relational, culturally safe, trauma informed social work service (at the centre of Figs. 1 and 2) or the ‘third space’.

### Creation of a Third Space for Social Work Practice

Based on Homi Bhabha’s concept of the third space, Aboriginal Health researchers Willis, Dwyer [25] argued for the creation of a third space in Australian healthcare. A space or ‘culture of interaction and confrontation’ between Aboriginal peoples and the colonising healthcare system requires *more than* respectful communication, modifying the environment to include Aboriginal artwork, changing behaviours or small scale innovations [25]. Here, we suggest the creation and use of the third space for hospital social work engagement and interventions, where power and difference are suspended. Both emotional and physical, this is a space where the social workers learn, and their ‘taken for granted’ understandings are challenged. An example is challenging the social worker’s concepts of ‘family’, ‘time’ and ‘next of kin.’ Creating a space where Aboriginal kinship and extended family systems are recognised and respected and Aboriginal health consumers are empowered to share their experiences and concerns. This will mean social workers will need time to listen deeply, establish trust to be able to identify who the Aboriginal health consumer recognises as their family and which family members are needed to be involved in decision-making about their care. It may for example involve reconceptualising their physical intervention space, as those members of the family who need to be there may outnumber the seats in the conventional family meeting room and require resources (e.g. digital or transport) to support family on country to be involved in decision-making and support. Further, some health issues must be treated as either men’s or women’s business [48]; when alerted to the need for gendered care, social workers need an authorising environment which supports the provision of this to Aboriginal health consumers.

In consultation with Aboriginal researchers, modifications were made to social work’s bio-psychosocial assessment tools and processes to move toward cultural engagement. In addition, transparency and involvement of family members in decision-making, as well as opportunities for social workers to co-work with Aboriginal Liaison Officers (ALO) or Aboriginal practitioners, were ways to manage their professional ethical commitments. These general ethical responsibilities include cultural safety and sensitivity and upholding standards of ethical conduct to prioritise the service users’ interests, with due respect to the rights of others and service user self-determination [20]. In cases of concern regarding the safety of the Aboriginal infant, co-working allows for the ALO to ensure the prioritisation of the Aboriginal mother’s self-determination and family involvement, while the social worker ensured the safety of the Aboriginal infant. Co-working and finding common ground allowed the social worker to begin to reconcile the tension between their understanding of Aboriginal culture with their institutional role. Reviewing the experience of co-working from all perspectives may serve to build evidence for culturally safe and trauma-informed care. While Stolen Generation provides a historical context for the distrust of social work, ongoing disproportionate rates of Aboriginal infant removals by social workers into state care have raised ongoing concerns about a new Stolen Generation [49]. The negative stereotypes of social work are further engrained by the inadequacies of the state (by proxy social workers) to implement the ‘Aboriginal Child Placement principle’ which prioritises the placement of Aboriginal children into kinship care with Aboriginal families [50]. Further demonstrating an ongoing disrespect for Aboriginal family structures. Change is required as the enduring impacts of colonisation are evident in the economic and health disparities for members of the Stolen Generation. When compared with non-Stolen Generation Aboriginal peoples, members of the Stolen Generation were found to be 1.8 times more likely to not be the owner of a home and 1.4 times more likely to have poor mental health and a severe or profound disability [51]. Author’s hope this research, where Aboriginal voices were listened to and changes to social work practices resulted, will serve to build trust with Aboriginal health consumers and begin to dismantle stereotypes.

The social workers in this study were aware of the role and importance of the engagement of ALOs within the hospital and their social work practice to enhance cultural safety and co-work with the ALOs. They were also aware there were only two, one male and one female, and the high demands on the ALO, which is not unusual in a hospital setting [47]. This reduced their capacity to co-work and offer culturally appropriate gendered care. So, while engagement with the ALO was considered important, it was also recognised that the social worker would on occasion need to ‘go at it alone’ without support from the ALO. This came with nervousness and a request for training support from the ALO, aligning with a study examining the relationship between social workers and hospital ALOs [47]. The relationship between the social workers and ALOs within hospital settings are increasingly considered highly important to build the cultural capacity of social workers [17–19]. All of these actions require ‘hospital time’ [52, 53]: time for social workers to engage and develop a trusting and respectful relationship with the Aboriginal health consumer; time to bring the right family members together; time to gain training from ALOs and time for ALOs to deliver training.

In addition, Aboriginal health consumers challenged western concepts of time relied upon within social work practice, in particular, the use of ‘past’ history as a measure of who and where they are now in the present. Concepts of time in Aboriginal communities have been described as circular and ‘here-now time’ [54] and constituted observationally and relationally [55], rather than linear in western conceptualisations of time. These challenges to concepts of time are also important in thinking about the westernised notion of ‘social work practice-time’ [53] and the time required to develop relationally based culturally safe environments in a busy, fast-paced hospital. Constraints on time are experienced by social workers due to the pressures from health administrators for social workers to meet ‘artificially prescribed’ hospital key performance indicators, for example, the number of patients they see, the constraints for the timeliness of the services provided and the constraints about having services in the community they can refer to who do not have waiting lists [53]. This limits the social workers’ ability to provide the services the Aboriginal health consumer needs.

Most hospital social workers articulated ‘quality care’ for Aboriginal health consumers aligned with what Aboriginal health consumers reported. The hospital social workers struggled with ‘how’ to realise, honour and respect Aboriginal culture and cultural practices and create a third space within the fast-paced hospital environment. In this local health network, the social workers were supported to engage in a culturally safe manner with Aboriginal health consumers by their social work manager, Allied Health Director and Aboriginal leadership within the hospital. Resulting from this study, the following impacts were realised contributing to the creation of a third space. The SW team developed a local social work RAP prioritising the need for the appointment of Aboriginal social workers, identifying specific areas for action such as placing reconciliation as an agenda item on the Social Work team meeting and ensuring an Aboriginal cultural focus at all meetings. The social work initial assessment tool/guide was modified to be more culturally sensitive, in consultation with Aboriginal researchers and social work staff engaged in training. The training involved an Aboriginal social worker upskilling staff on the use of ecomaps and genograms with Aboriginal family structures, and an Aboriginal health consumer shared their experiences of social work in acute care. A handheld tool titled ‘Who’s your mob?’ was designed by an Aboriginal artist. This tool supports social workers to engage with the Aboriginal consumer in a conversation about ‘who their mob is?’, inviting the consumer to show on a map of their important family locations. This then often leads to explaining important cultural connections and family-specific care roles. Opportunities for co-working with Aboriginal health practitioners are being used to dismantle stereotypes and work to build greater trust with Aboriginal health consumers. In addition, the authorising environment surrounding the social workers was supportive of providing high-quality care to Aboriginal health consumers. For example, ‘Action 1.21: Improving cultural competency’ in the NSQHS for Aboriginal and Torres Strait Islander health states: ‘providing a supportive environment and clear processes for the workforce to explore the cultural needs of Aboriginal and Torres Strait Islander patients’ [4]. Impacts from this study included the identification of a social work interview room with windows to the outdoors (views of trees) and access to the outdoors providing a less clinical environment. Further, social work commissioned an Aboriginal artist to design a decal for the internal window of the social work interview room, providing a more inviting inter-cultural space. These changes signal shifts to decolonising social work practices within the confines of the hospital and dismantling cultural hegemony. Aboriginal health consumer feedback is being monitored through patient-reported experience measures and monitoring of uptake of social work services by Aboriginal health consumers and reduced complaints.

The only outlier in relation to the ‘supportive environment’ are the pressures placed on social work staff by hospital administrators to meet or reduce the allocated length of stay for the health condition and ensure timely discharge from the hospital [11, 56]. Hospital’s funding and payment models influence and shape the organisation and operation of hospital services [57]. Social workers reported feeling pressure from hospital administrators regarding managing caseloads, the number of people they have seen and the provision of a timely service response. Reconceptualising notions of time within healthcare [52, 53], the health services funding model could make provisions for ‘cultural time’ or the recognition of the additional time required when working cross-culturally to establish and build relationships in healthcare. Social workers experienced cognitive dissonance as they struggled between knowing how they ‘should’ engage with Aboriginal health consumers to provide a quality social work service and what they know they can provide within the administrative confines of the fast-paced busy hospital. Notions of both ‘timely’ and ‘quality’ social work service responses are at ‘loggerheads’, and both need to be reconceptualised with a cultural lens, such that there is a recognition through all structures within the hospital that any social work engagement with Aboriginal health consumers in hospitals will require more time.

As with all research, there were limitations in the conduct of this study. This study was not a representative sample and, at best, might be generalisable to other similar Australian hospital contexts within low socio-economic status communities. To protect participant anonymity and privacy and reduce participant burden, limited demographic data was collected. Demographic data was limited to cultural background and gender, reducing the capacity for intersectional analysis. In addition, the small sample size of Aboriginal health consumers recruited through Aboriginal health services was an additional limitation. Even though it was a small sample, there were common experiences and so reached saturation [38]. It is too early to evaluate the success of the implementation of the third space in this context; however, early feedback has included positive feedback through health service patient-reported experience measures. For example, an Aboriginal client provided feedback to an Aboriginal liaison officer about how she observed the SW to be sensitive to her cultural needs. This study was conducted during COVID which both delayed and reduced momentum. Natural disasters also impacted the conduct of this study, with flooding closing the Aboriginal health service on one of our data collection days. A strength of the research was the involvement of Aboriginal peoples throughout from conception to design, data collection, analysis, reporting and dissemination.

## Conclusions

Aboriginal health consumers articulated the need for change to how hospital social workers engage with them. They called for social workers to ask about them, their connection to culture, their Nation or group and their family. This process of inquiry will help to build connections and establish relational practice. They called for the recognition of gendered care, the valuing of family involvement and the need for an understanding of who they are now, not who they may have been in their past. To do this, Aboriginal health consumers recognised the value of the RAP and the need for employment of Aboriginal staff. Social workers spoke of knowing the impacts of colonisation, the role the social work profession played and their fear of offending Aboriginal health consumers in their practice. They questioned how to put this knowledge into action. They spoke of the tensions managing the demands of working in a busy hospital environment and being able to provide relational, patient-centred, culturally safe, trauma-informed care in the fast-paced and time-pressured hospital environment. The creation of a third space for social work practice including the development of a Who’s your mob? tool provided opportunities for action such as challenging personal and professional power and engaging in decolonisation of taken-for-granted social work practices and respecting Aboriginal ways of knowing, doing and being.

## Data Availability

The data in this study cannot be shared openly. This is to protect study participant privacy and ensure that Aboriginal data sovereignty is respected. Data in this study was collected and handled in a culturally safe and respectful way, recognising Aboriginal peoples’ rights to control their own data.

## Footnotes

1 In this paper we use the term ‘Aboriginal’, unless a source refers directly to ‘Indigenous’ or ‘Aboriginal and Torres Strait Islander’. This research was conducted in South Australia where the Indigenous peoples of the land self-identify as Aboriginal, hence we use the term in consideration of the preference of the Aboriginal people involved in this research.
